# In vivo quantification of neurofibrillary tangles with [^18^F]MK-6240

**DOI:** 10.1186/s13195-018-0402-y

**Published:** 2018-07-31

**Authors:** Tharick A. Pascoal, Monica Shin, Min Su Kang, Mira Chamoun, Daniel Chartrand, Sulantha Mathotaarachchi, Idriss Bennacef, Joseph Therriault, Kok Pin Ng, Robert Hopewell, Reda Bouhachi, Hung-Hsin Hsiao, Andrea L. Benedet, Jean-Paul Soucy, Gassan Massarweh, Serge Gauthier, Pedro Rosa-Neto

**Affiliations:** 1 Translational Neuroimaging Laboratory, The McGill University Research Centre for Studies in Aging, 6825 LaSalle Boulevard, Verdun, QC H4H 1R3 Canada; 2 Montreal Neurological Institute, 3801 University Street, Montreal, QC H3A 2B4 Canada; 3 Translational Biomarkers, Merck & Co., Inc., 770 Sumneytown Pike, West Point, PA 19486 USA; 4 Douglas Hospital, McGill University, 6875 La Salle Blvd—FBC room 3149, Montreal, QC H4H 1R3 Canada

**Keywords:** Tau positron emission tomography, Neurofibrillary tangles, Alzheimer’s disease

## Abstract

**Background:**

Imaging agents capable of quantifying the brain’s tau aggregates will allow a more precise staging of Alzheimer’s disease (AD). The aim of the present study was to examine the in vitro properties as well as the in vivo kinetics, using gold standard methods, of the novel positron emission tomography (PET) tau imaging agent [^18^F]MK-6240.

**Methods:**

In vitro properties of [^18^F]MK-6240 were estimated with autoradiography in postmortem brain tissues of 14 subjects (seven AD patients and seven age-matched controls). In vivo quantification of [^18^F]MK-6240 binding was performed in 16 subjects (four AD patients, three mild cognitive impairment patients, six healthy elderly individuals, and three healthy young individuals) who underwent 180-min dynamic scans; six subjects had arterial sampling for metabolite correction. Simplified approaches for [^18^F]MK-6240 quantification were validated using full kinetic modeling with metabolite-corrected arterial input function. All participants also underwent amyloid-PET and structural magnetic resonance imaging.

**Results:**

In vitro [^18^F]MK-6240 uptake was higher in AD patients than in age-matched controls in brain regions expected to contain tangles such as the hippocampus, whereas no difference was found in the cerebellar gray matter. In vivo, [^18^F]MK-6240 displayed favorable kinetics with rapid brain delivery and washout. The cerebellar gray matter had low binding across individuals, showing potential for use as a reference region. A reversible two-tissue compartment model well described the time–activity curves across individuals and brain regions. Distribution volume ratios using the plasma input and standardized uptake value ratios (SUVRs) calculated after the binding approached equilibrium (90 min) were correlated and higher in mild cognitive impairment or AD dementia patients than in controls. Reliability analysis revealed robust SUVRs calculated from 90 to 110 min, while earlier time points provided inaccurate estimates.

**Conclusions:**

This evaluation shows an [^18^F]MK-6240 distribution in concordance with postmortem studies and that simplified quantitative approaches such as the SUVR offer valid estimates of neurofibrillary tangle load 90 min post injection. [^18^F]MK-6240 is a promising tau tracer with the potential to be applied in the disease diagnosis and assessment of therapeutic interventions.

## Background

In vivo quantification of neurofibrillary tangles constitutes a new challenge in the field of Alzheimer’s disease (AD) imaging research. It is expected that reliable imaging agents that are able to accurately quantify tangles in the human brain will complement the information provided by the existing amyloid-β tracers, allowing a more precise staging of AD. In addition, these imaging agents may prove to be crucial for the enrichment of clinical trial populations with tau-positive individuals and for monitoring the efficacy of disease-modifying interventions.

Over the last few years, three classes of tau tracers have appeared as candidates to selectively measure neurofibrillary tangles in the living human brain: the derivative of pyrido-indole ([^18^F]AV1451) [1, 2], the derivatives of aryquinoline ([^18^F]THK5117, [^18^F]THK5317, and [^18^F]THK5351) [3, 4], and the derivative of phenyl/pyridinyl-butadienyl-benzothiazoles/benzothiazolium ([^11^C]PBB3) [5]. Although these tracers have shown affinity to neurofibrillary tangles, compelling evidence suggests that off-target binding heavily influences the signal of some of them, even in cortical brain regions that are considered a target for AD. For example, in a recent in vivo study, we have shown that the binding of [^18^F]THK5351 in regions including the cingulate, temporal, and inferior parietal cortices is strongly driven by MAO-B availability [6]. Similarly, in vitro evidence suggests that MAO-A may influence the signal of [^18^F]AV1451 [7] and that [^11^C]PBB3 may bind to α-synuclein pathology [8]. Furthermore, most of these tracers have shown high binding in the striatum, which is not a region where the histopathological studies show a high density of tangles in AD [9]. Thus, a tau-imaging agent with low brain off-target binding remains an unmet need in the field of AD research.

The pyrrolopyridinyl isoquinoline amine derivative [^18^F]MK-6240 has been recently developed by Merck. [^18^F]MK-6240 is a tracer with a subnanomolar affinity and high selectivity for neurofibrillary tangles that showed excellent physicochemical properties in a preclinical observation [7]. [^18^F]MK-6240 has shown characteristics with the potential to fulfill the criteria for a promising new-generation tau tracer, such as reduced brain off-target binding, fast brain penetration and kinetics, and the absence of brain permeable metabolite. Here, we aim to quantify [^18^F]MK-6240 using gold standard methods with metabolite-corrected arterial input function. In addition, we aim to validate simplified methodologies that are capable of bypassing the need for invasive arterial sampling in young and elderly cognitively healthy (CN) individuals, mild cognitive impairment (MCI) patients, and AD patients.

## Methods

### [^18^F]MK-6240 autoradiography

In vitro autoradiography with [^18^F]MK-6240 was conducted in postmortem brain samples of patients with antemortem diagnosis of AD (Consortium to Establish a Registry for Alzheimer Disease (CERAD) positive) [10]) and CN subjects (CERAD negative) obtained from the Douglas-Bell Canada Brain Bank with the approval of the Brain Bank’s scientific review and Douglas Institute’s research ethics boards. In total, seven AD patients and seven CN individuals were studied. Six AD and six CN individuals had the cerebellum, hippocampus, and prefrontal regions assessed (one AD p and one CN individual did not have viable hippocampal sections). One AD patient and one CN individual had their whole hemisphere sections assessed. The postmortem delay ranged from 8.5 to 18.25 h and from 17.25 to 21.25 h in AD patients and CN individuals, respectively. Briefly, flash-frozen tissues were cut into 20 m thick sections and thawed on coated microscope slides using a freezing sliding microtome (Leica CM3050 S) at − 15 °C. The samples were then air-dried, warmed up to room temperature, and preincubated with 1% bovine serum albumin in a phosphate-buffered saline solution (pH 7.4) for 10 min to remove endogenous ligands. These samples were then once more air-dried and incubated with 20.4 MBq of [^18^F]MK-6240 in 600 ml of phosphate-buffered saline solution for an additional 150 min. Subsequently, the tissues were dipped three times in the phosphate-buffered solution and, dipped in distilled water at 4 °C and dried under a stream of cool air. Finally, the samples were exposed to a radioluminographic imaging plate (Fujifilm BA SMS2025) for 20 min and the activity in photostimulated luminescence units per mm^2^ was calculated using ImageGauge software 4.0 (Fujifilm Medical Systems, Inc.). The activity in each individual’s brain region was measured in three equidistant regions of interest (ROIs) placed by an experimenter blind to clinical diagnosis. Then, an averaged value of these three ROIs was used as the final region uptake. In order to correct for background noise and nonspecific activity, this uptake was normalized to the individual’s cerebellar gray matter uptake. A *t* test assessed the difference in uptake across diagnostic groups. Further details regarding the in vitro autoradiography methods may be found elsewhere [11].

### Participants

AD and MCI patients and young and aged CN individuals were recruited after extensive clinical assessments at the McGill University Research Centre for Studies in Aging. All participants underwent a detailed neuropsychological evaluation including the Clinical Dementia Rating (CDR) and Mini-Mental State Examination (MMSE) scales, an amyloid-PET scan with [^18^F]AZD4694 in order to assess the presence of brain AD pathophysiology, and [^18^F]MK-6240. CN individuals had no subjective or objective cognitive impairment and a CDR of 0. MCI patients had a CDR of 0.5, subjective and objective memory impairments, and essentially normal activities of daily living. AD dementia patients had CDR equal to or greater than 1 and met the National Institute on Aging and the Alzheimer’s Association criteria for probable AD [12]. None of the individuals met the criteria for other neuropsychiatric disorders.

### Radiosynthesis

[^18^F]Fluoride was produced via the ^18^O(p,n)^18^F reaction in a water target. The target filling was transferred with a steady stream of argon into a septum-closed vial inside a hot cell, where the synthesis of [^18^F]MK-6240 took place. After delivery of the radioactivity, the module transferred the fluoride anion onto a quaternary methyl amine (QMA) cartridge where it was retained, and the target water was transferred into a collection vial for recycling. [^18^F]Fluoride was then eluted off the QMA cartridge and into the reactor with a solution of 1.35 ml of acetonitrile containing 15 ± 1 mg tetraethyl ammonium carbonate. The solution was then evaporated to dryness repeatedly with additional acetonitrile at a temperature of 95 °C, a stream of nitrogen, and reduced pressure. After 15 min, a solution of 1 mg of the MK-6240 precursor in 1 ml dimethyl sulfoxide (DMSO) was added to the reactor and was heated stepwise to 150 °C for about 20 min. During this step, the deprotected final product [^18^F]MK-6240 was formed. The reactor was then cooled to 70 °C, and 1.5 ml of high-pressure liquid chromatography (HPLC) solvent (20 mM sodium phosphate/CH3CN, 78/22) was added. The resulting mixture was transferred into the injector loop of the HPLC system and purified on a Luna C18 250 mm × 10 mm 5-μM HPLC column (Phenomenex Inc.), with a flow of 5 ml/min. The desired product eluted at a retention time of about 22-24 min. The product peak was collected with a 30-ml syringe containing 18 ml of water and 50 μl of ascorbic acid. The solution was passed through a C18 Sep-Pak cartridge (Waters Corp.). The cartridge was washed with an additional 10 ml of water. The product was then eluted from the cartridge into a round flask with 5 ml of ethanol. The flask was sealed off with a vacuum piece attached to a vacuum pump, and a water bath was raised to insert the flask into hot water. The HPLC solvent was evaporated to dryness under reduced pressure, and approximately 5 ml of ethanol was added to the flask via a three-way stopcock and a line attached to the vacuum piece. The ethanol was evaporated to dryness, and if any solvent remained visible, another 5 ml of ethanol USP (United States Pharmacopeia) was added and evaporated again. The dried [^18^F]MK-6240 was then dissolved in 0.5 ml of ethanol and then 9.5 ml of sterile saline, and transferred into a 10-ml syringe with an attached needle. The needle was then removed and the syringe was inserted into the female Luer lock of a sterile filter, which is part of a preassembled bulk vial. Finally, the [^18^F]MK-6240 was sterile filtered into the product vial. [^18^F]MK-6240 had an average injected dose of 241 (standard deviation (SD) = 23) MBq with a specific activity at the time of injection of 629 (SD = 330) GBq/μmol. [^18^F]AZD4694 was synthesized according to the previously published literature [13], with an average injected dose of 237 (SD = 19) MBq and an average specific activity at the time of injection of 325 (SD = 480) GBq/μmol.

### PET acquisitions

PET scans were performed using the Siemens high-resolution research tomograph (HRRT) PET scanner. [^18^F]MK-6240 images were acquired dynamically and uninterruptedly in list mode files between 0 and 180 min after the intravenous bolus injection of the tracer. [^18^F]MK-6240 scans were reconstructed using an ordered-subsets expectation maximization (OSEM) algorithm on a 4D volume with 52 frames (12 × 6 s, 6 × 18 s, 4 × 30 s, 5 × 60 s, 5 × 120 s, 8 × 300 s, and 12 × 600 s) [14]. [^18^F]AZD4694 images were acquired 40–70 min after the intravenous bolus injection of the tracer, and the scans were reconstructed with the same OSEM algorithm on a 4D volume with three frames (3 × 600 s). A 6-min transmission scan was conducted with a rotating ^137^Cs point source at the end of each dynamic acquisition for attenuation correction. All images were subsequently corrected for dead time, decay, and both random and scattered coincidences. A head holder was used to reduce head motion during the scan time. In addition, possible movements during the scanning procedure were corrected using a coregistration-based method that performs frame realignment and compensates for emission–transmission mismatches [15].

### Image processing

All participants had an anatomical 3D T1-weighted magnetic resonance imaging (MRI) scan (3 T Siemens). The image analyses were performed using the Medical Image NetCDF software toolbox (www.bic.mni.mcgill.ca/ServicesSoftware/MINC). In brief, the T1-weighted images were corrected for field distortions, segmented, nonuniformity corrected, and processed using the CIVET pipeline [16]. Subsequently, the T1-weighted images were linearly registered to the MNI reference template space [17], whereas the PET images were automatically coregistered to the individual’s MRI space. Then, the final PET linear registration was performed using the transformations obtained from the MRI to MNI linear template and the PET to T1-weighted native image. PET images were then spatially smoothed to achieve a final resolution of 8-mm full-width at half maximum. ROIs were obtained from the MNI nonlinear ICBM atlas and subsequently reoriented to the individual’s linear space [18]. The ROIs were tailored from the frontal, medial prefrontal, orbitofrontal, precuneus, anterior (ACC) and posterior cingulate (PCC), lateral and mediobasal temporal, inferior parietal, parahippocampus, hippocampus, insula, occipitotemporal, occipital pole, and cerebellar cortices as well as from the striatum, the pons, and the telencephalon white matter (cerebellar white matter not included). Subsequently, the ROIs were applied to the dynamic PET frames to obtain the time–activity curve data. The parametric images and the ROI standardized uptake value ratios (SUVRs) were measured for multiple different scan time frames and were generated using the cerebellar gray matter as the reference. Amyloid-PET positivity was determined visually by two raters blind to clinical diagnosis. Further information regarding the imaging methods pipeline may be found elsewhere [19, 20].

### [^18^F]MK-6240 metabolism

During the [^18^F]MK-6240 scans, arterial blood samples were collected with an automatic blood sampling system (Swisstrace GmbH) throughout the full scanning procedure with a pump flow rate of 5 ml/min between 0 and 10 min and 0.65 ml/min between 11 and 180 min. Additional 5 ml samples for metabolite correction were collected manually at 5, 10, 20, 40, 60, 90, 120, and 180 min. A cross-calibrated gamma well counter (Caprac, Inc.) was used to measure the radioactivity in the whole blood and in the plasma. Briefly, the manual samples were collected with a 5-ml heparinized syringe and centrifuged at 4000 rpm for 5 min at 4 °C. Then, the plasma was separated from the blood cells, and 1 ml was diluted with 1 ml of acetonitrile. The samples were vortexed and once more centrifuged at 4000 rpm for 5 min at 4 °C. Then, the supernatant was separated and filtered using a Millipore GV 13-mm-diameter filter. This supernatant plasma was injected into the HPLC system (Waters 1525 Binary HPLC pump; Waters Corp.), connected to a UV/visible detector (Waters 2489 UV/Visible Detector) and a coincidence detector (Bioscan, Inc.), with a flow rate of 1 ml/min. This dedicated radio-HPLC system used an isocratic method with a C18 analytical column (XTerra MS C18 column, 5 μm, 4.6 mm 250 mm) and a mobile phase consisting of 55% sodium acetate and 45% acetonitrile in order to provide the parent-to-metabolite ratio. The radioactivity estimated with the radio-HPLC system was denoised to reduce the instability generated by its low levels in the later time frames. After background correction and cross-calibration with the PET scanner, the blood activity was multiplied by both the plasma-to-whole blood and the parent compound fractions in order to derive the metabolite-corrected plasma input function.

### [^18^F]MK-6240 kinetic modeling

The kinetic modeling was performed using KinFit (version 1.7) and PMOD (version 3.8) software. Using the metabolite-corrected plasma input function, the kinetic parameters of [^18^F]MK-6240 were initially quantified using a reversible two-tissue compartment model with four parameters (2T-CM4k), assuming rapid kinetics between free and nonspecifically bound tracer. For the 2T-CM4k, the total distribution volume (V_T_) was measured as V_T_ = (*K*_1_ / *k*_2_).(1 + *k*_3_ / *k*_4_) (ml/cm^3^), and the binding potential (BP) was directly measured as *k*_3_ / *k*_4_. In this equation, *K*_1_ (ml/cm^3^/min) and *k*_2_ (1/min) represent the transport rates for the influx and efflux of the tracer across the blood–brain barrier, and the rate constants *k*_3_ (1/min) and *k*_4_ (1/min) represent the exchange from the nondisplaceable (ND; free and nonspecific tracer) to the specific binding compartment and return, respectively [21]. In addition, a one-tissue compartment model (1T-CM), an irreversible 2T-CM with three fitted parameters (2T-CM3k), and a Logan linear graphical method were performed [22]. The distribution volume ratio (DVR) was calculated by dividing the V_T_ from a target region by the distribution volume of a region assumed to contain only free and nonspecifically bound tracer (V_ND_; cerebellar gray matter). We assumed a fractional tissue blood volume of 5% for the aforementioned models. The simplified reference tissue model (SRTM) and the reference Logan method, both using the cerebellar gray matter as the reference, were also fitted in order to obtain DVRs (BP_ND_ + 1) [23–25]. A *k*_2_′ value was estimated for each individual with the SRTM and used in the reference Logan method. The model’s goodness of fit was assessed with *R*^2^ and *F* test, while the Akaike information criterion (AIC) was used to compare the models [26]. A *t* test compared AIC values from different quantification methods. We tested the similarity between models with regression analysis based on all ROIs.

### Determination of the optimal time window for the SUVR calculation

[^18^F]MK-6240 secular and peak equilibria were used to guide the determination of the first time point used for the SUVR calculation. The shortest scan duration without compromising reliability was determined by analyzing the stability of SUVRs calculated using different time durations. Finally, we validated SUVR estimates against the gold standard 2T-CM4k with the metabolite-corrected plasma input function.

The time to reach the secular equilibrium was assumed as the point at which the curves representing the ratio of the target-to-reference region reached a plateau [27]. Similarly, the peak equilibrium was calculated using specific binding curves estimated as the difference between the target and the reference regions [13], where the equilibrium was defined when *d*Cb(specific binding) / *d*(*t*) = 0. The target region used in the analyses was a composite value from the regions with the highest ligand uptake (precuneus, posterior cingulate, inferior parietal, and lateral temporal cortices), whereas the reference region was the cerebellum gray matter. The time–activity curves were normalized for the injected tracer dose and the individual’s weight.

In order to assess the optimal scan duration, we tested the stability of the coefficient of variation (CV) and the intraclass correlation coefficient (ICC) in SUVRs obtained with progressive longer durations (10, 20, 30, 40, 50, and 60 min) [28, 29]. The 95% confidence interval (CI) determined the absence of change in the CV and the ICC among the different SUVR durations. For each SUVR time duration, a subject’s CV was determined as the average of CVs obtained within each ROI. ICCs were determined between SUVR time durations using all individuals’ ROIs. In addition, ICCs were calculated between SUVR and 2T-CM4k DVR and BP (*k*_3_ / *k*_4_) estimates.

Finally, we estimated the slope, intercept, and *R*^2^ between 2T-CM4k and SUVRs measured over the 52 reconstructed time frames in order to ascertain the points with an optimal equivalence between these two methods.

## Results

**Fig. 1:**
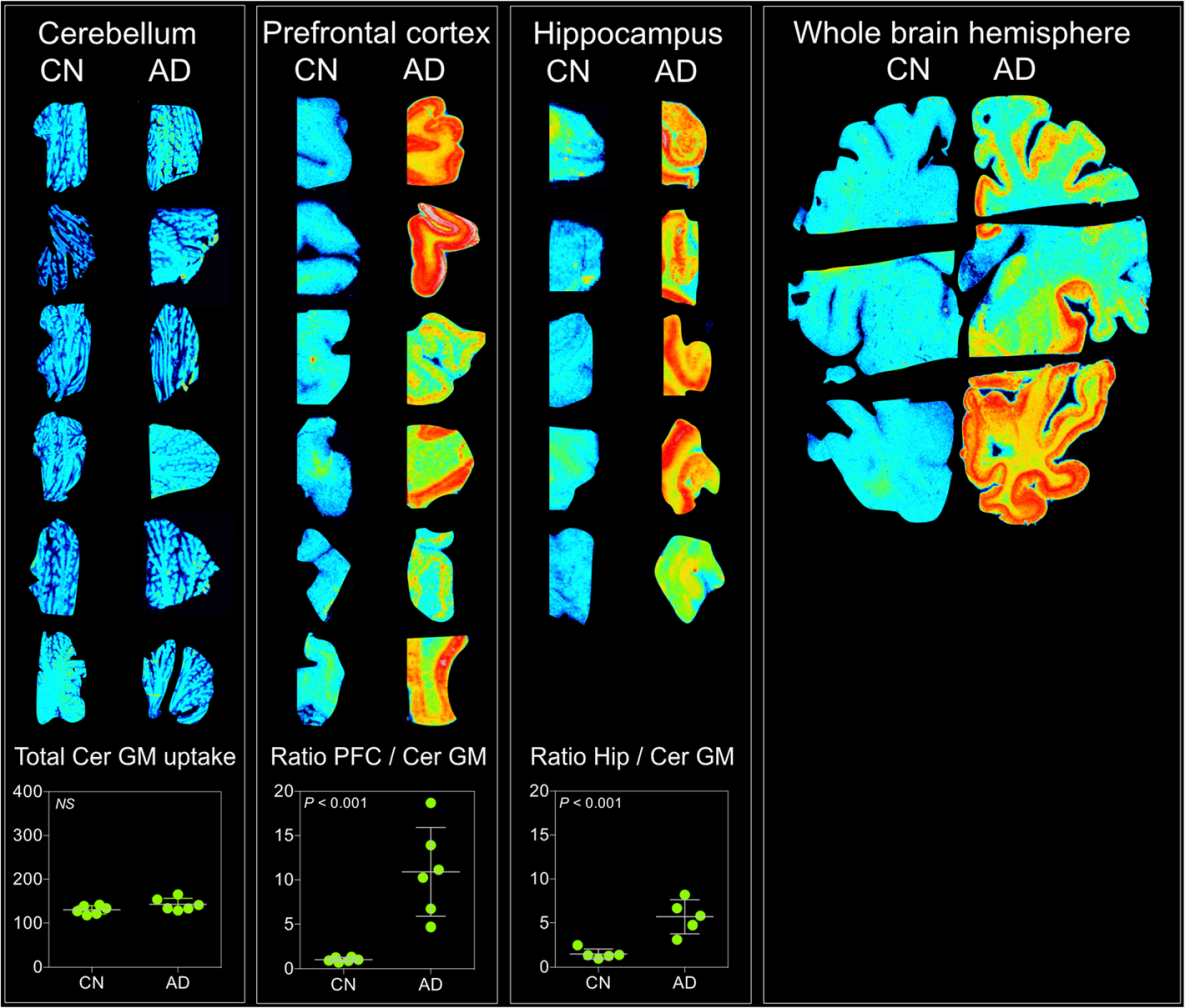
[^18^F]MK-6240 autoradiographs of postmortem brain tissues of AD and CN individuals. In vitro autoradiography of postmortem brain tissue from prefrontal (PFC) and hippocampal (Hip) cortices as well as cerebellum and whole hemisphere of Alzheimer’s disease (AD) patients and cognitively healthy (CN) individuals. Similar total uptake was found in cerebellar gray matter (Cer GM) of CN and AD individuals (*P* = 0.2). AD patients had higher relative uptake (ratio with Cer GM) than CN individuals in PFC and Hip cortices (*P* < 0.001). Total uptake was measured as photostimulated luminescence units per mm^2^. NS not significant

In vitro [^18^F]MK-6240 autoradiography was performed in postmortem brain tissues from seven AD patients (mean age of death 72.5 (SD = 10) years, three males) and seven CN individuals (mean age of death 70 (SD = 13) years, three males). The total brain mass was 24% lower in AD patients (mean 977 (SD = 195) g) than CN subjects (mean 1221 (SD = 112) g) (*P* = 0.03). Autoradiographs showed greater [^18^F]MK-6240 uptake in AD patients than in CN individuals in brain regions that were expected to contain tangles such as prefrontal and hippocampal cortices, whereas similar uptake was found in the cerebellar gray matter (*P* = 0.2) (Fig. 1).

In vivo dynamic [^18^F]MK-6240 acquisitions were performed in 16 individuals (four AD patients, three MCI patients, six elderly CN individuals, and three young CN individuals). The demographics and key characteristics of the in vivo population are summarized in Table 1.

**Table 1:** Demographics and key characteristics of the in vivo population

	Young CN individuals	Elderly CN individuals	MCI patients	AD patients
*N*	3	6	3	4
Age (years), mean (SD)	22.3 (1.5)	67.8 (8.5)	69 (3.4)	60.7 (6)
Male, *n* (%)	2 (67)	5 (83)	2 (67)	3 (75)
MMSE, mean (SD)	30 (0)	29.5 (0.5)	25 (2.6)	17.3 (5.8)
*APOE ε4*, *n* (%)	0 (0)	4 (67)	1 (33)	2 (50)
Amyloid-PET positive, *n* (%)	0 (0)	1 (17)	3 (100)	4 (100)

*AD* Alzheimer’s disease, *CN* cognitively healthy, *MCI* mild cognitive impairment, *MMSE* Mini-Mental State Examination, *PET* positron emission tomography, *SD* standard deviation

**Fig. 2:**
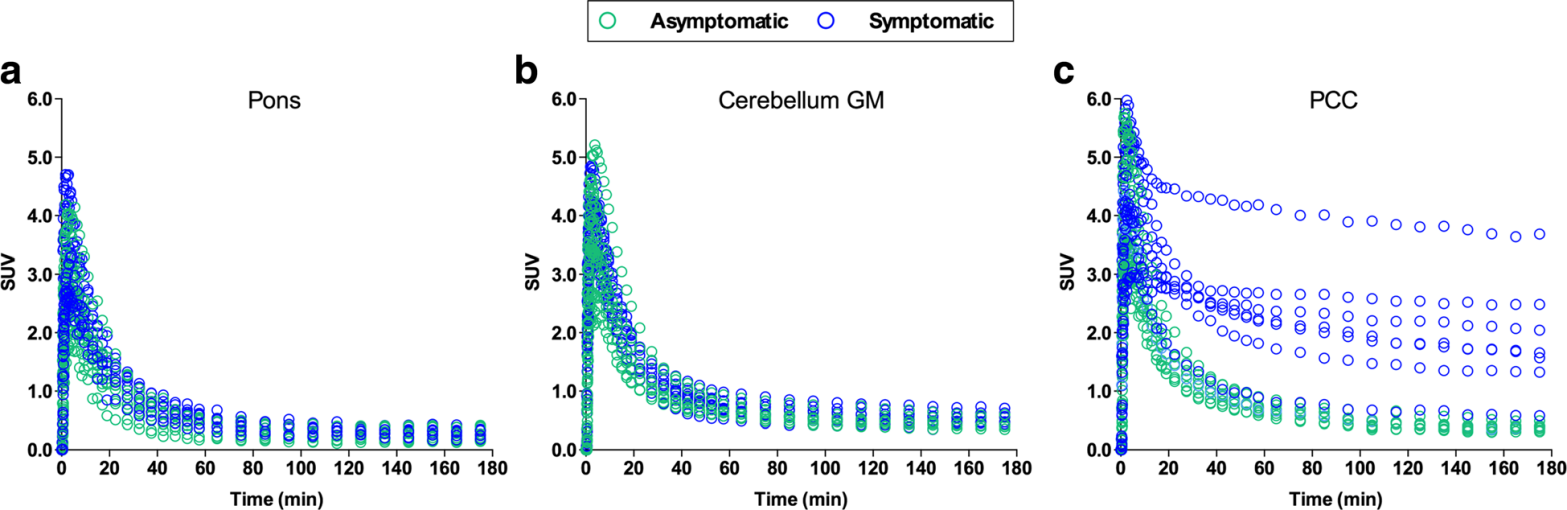
Regional time–activity curves from selected brain regions for all participants. Regional standardized uptake value (SUV) time–activity curves of [^18^F]MK-6240 in pons (**a**), cerebellar gray matter (GM) (**b**), and posterior cingulate cortex (PCC) (**c**) for all participants. Green dots represent young and elderly cognitively healthy individuals, blue dots represent MCI and Alzheimer’s disease patients

Time–activity curves revealed that the radioactivity appeared rapidly in the brain with an SUV peak between 2 and 5 min after the [^18^F]MK-6240 injection. In young and elderly CN individuals, the time–activity curves had a uniform rapid washout and were similar in target and reference regions. In AD and MCI patients, the radioactivity showed a slower clearance in the regions that were expected to contain high concentrations of neurofibrillary tangles. Regional time–activity curves from selected brain regions of all individuals of the population are presented in Fig. 2.

**Fig. 3:**
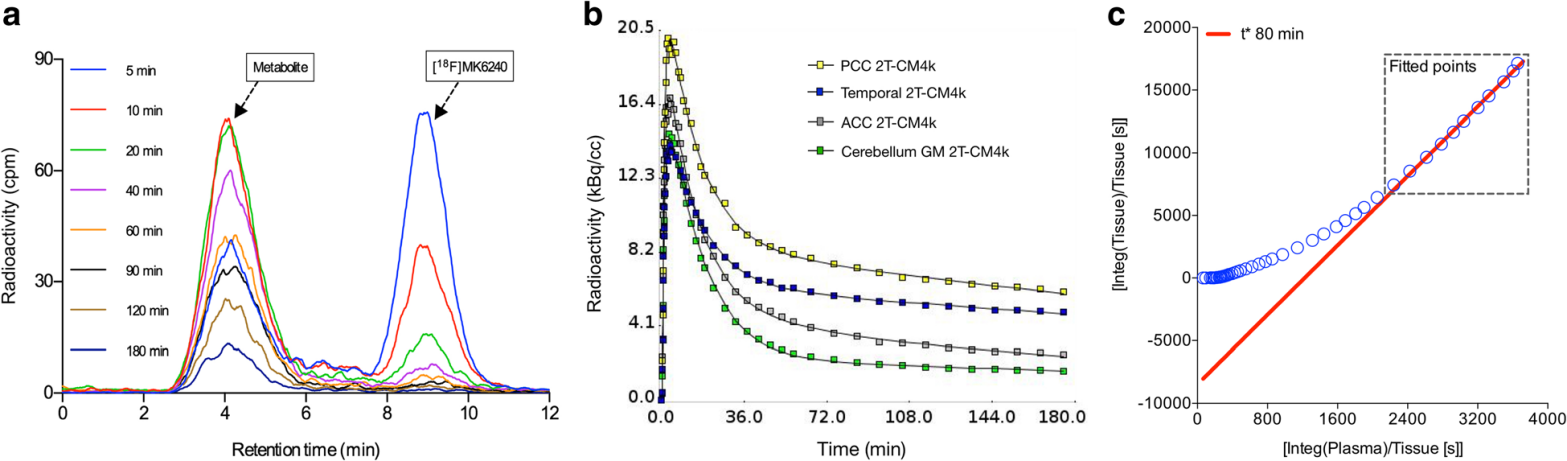
Chromatography, model compartmentalization, and data fit of [^18^F]MK-6240. **a** Chromatogram showing parent compound and metabolite of [^18^F]MK-6240 in counts per minute (cpm) for representative AD participant. **b** Reversible two-tissue compartment model with four parameters (2T-CM4k) fit in time–activity curves from posterior cingulate (PCC), temporal, and anterior cingulate (ACC) cortices, as well as cerebellar gray matter (GM) of representative mild cognitive impairment (MCI) individual. **c** Logan graphical plot became linear approximately 80 min after injection in PCC of representative MCI individual

Plasma analysis revealed that [^18^F]MK-6240 has rapid clearance from the blood. The radio-metabolite analysis suggested only one metabolite, which is more polar than the parent compound. The metabolite peak was identified in the plasma eluting with a retention time of 4 min, whereas the parent compound appeared at 8 min. After 10 min, approximately 70% of the parent compound was metabolized (Fig. 3a).

The time–activity curves were well described by the 2T-CM4k across individuals and brain regions (Fig. 3b). The 2T-CM4k (AIC mean 11.39 (SD = 33)) provided a better fit than the 2T-CM3k (AIC mean 69.98 (SD = 45)) visually and statistically across subjects and regions (*P* < 0.0001, *t* test). The kinetic parameters derived from the preferred 2T-CM4k are presented in Table 2. The cerebellar gray matter fitted the 2T-CM4k better, whereas the pons showed no difference in AIC between the fits with the 2T-CM4k and the 1T-CM. The Logan graphical model became linear approximately 80 min after the tracer injection in high binding regions (Fig. 3c).

**Table 2:** Kinetic parameters obtained with the 2T-CM4k

Region	*K*_1_	*k*_2_	*k*_3_	*k*_4_	V_T_	*K*_1_/*k*_2_	*k*_3_/*k*_4_	AIC	*R* ^2^
Asymptomatic individuals									
Cer GM	0.327 (0.08)	0.235 (0.00)	0.025 (0.03)	0.046 (0.04)	2 (0.1)	1.39 (0.36)	0.41 (0.3)	– 11 (2.8)	0.99 (0.00)
PCC	0.343 (0.03)	0.237 (0.04)	0.021 (0.01)	0.044 (0.01)	2.2 (0.1)	1.48 (0.42)	0.56 (0.52)	– 33.7 (4.3)	0.99 (0.00)
Symptomatic individuals									
Cer GM	0.230 (0.02)	0.125 (0.02)	0.009 (0.01)	0.01 (0.00)	3.4 (0.8)	1.88 (0.37)	0.86 (0.5)	– 2.0 (55)	0.99 (0.00)
PCC	0.246 (0.08)	0.099 (0.01)	0.052 (0.03)	0.01 (0.00)	14.2 (5.6)	2.45 (0.56)	5.11 (2.6)	– 3.7 (17)	0.99 (0.00)

Mean (standard deviation) of kinetic parameters obtained with the reversible two-tissue compartment model with four parameters (2T-CM4k) in the cerebellar gray matter (Cer GM) and posterior cingulate cortex (PCC) of asymptomatic (young and elderly cognitively healthy) and symptomatic (mild cognitive impairment and Alzheimer’s disease) individuals who underwent arterial sampling

*AIC* Akaike information criterion, *K*_1_ (ml/cm^3^/min) transport rate for influx of tracer across blood–brain barrier, *k*_2_ (1/min) transport rate for efflux of tracer across blood–brain barrier, k_3_ (1/min) rate constant for exchange from nondisplaceable (free and nonspecific tracer) to specific binding compartment, k_4_ (1/min) rate constant for exchange from specific binding compartment to nondisplaceable

**Fig. 4:**
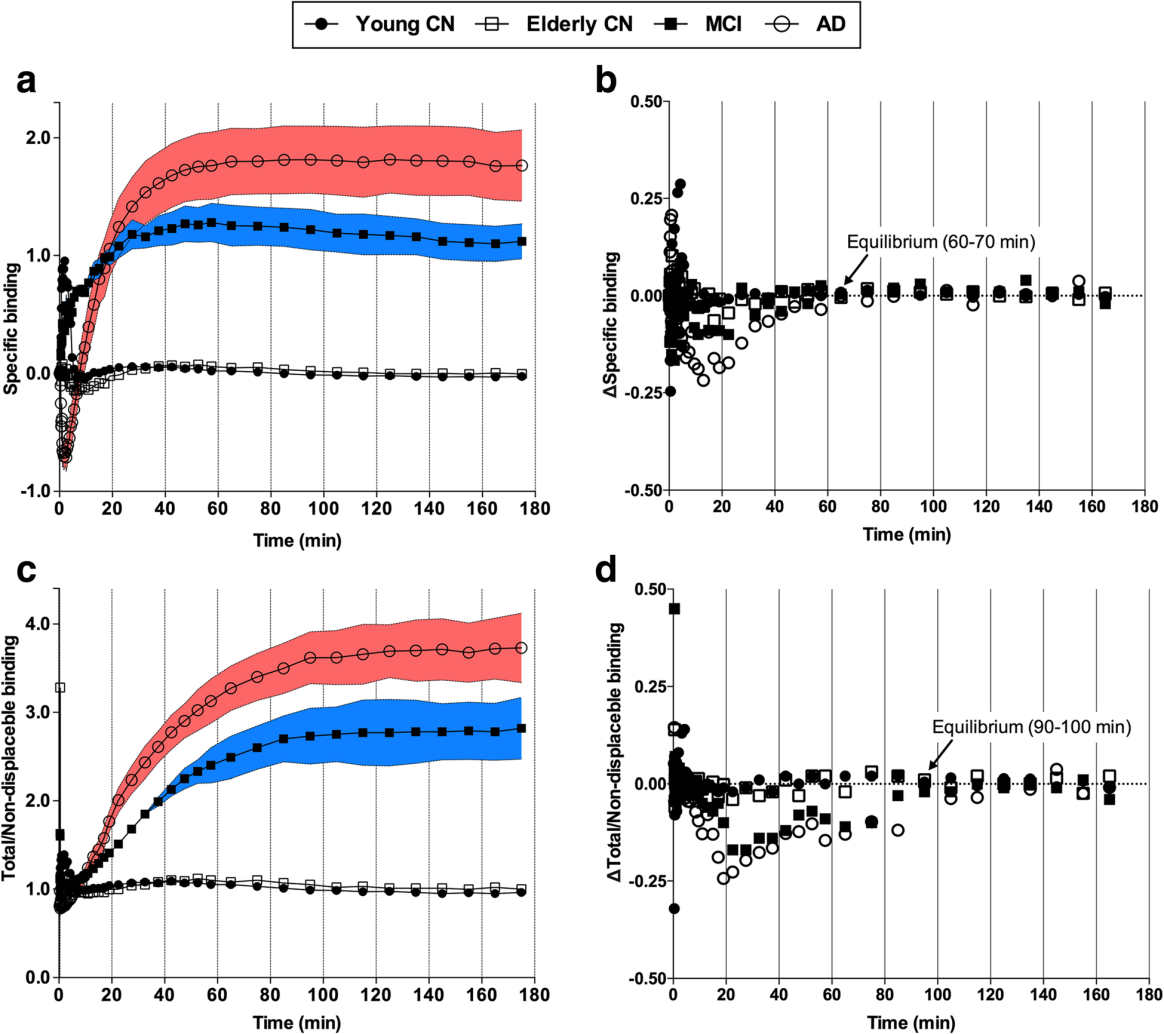
[^18^F]MK-6240 uptake reaches equilibrium during scan time. Curves show mean of specific (**a**) and total/nondisplaceable (ND) (**c**) binding across diagnostic groups over different scan acquisition time points and area between bars represents standard error of the mean. Variation (Δ) of specific (**b**) and total/ND (**d**) binding calculated as difference between averaged uptake value in a given time point to averaged uptake value in subsequent time point. Variation of specific and total/ND binding approached 0 in frames starting at 60 and 90 min for both mild cognitive impairment (MCI) and Alzheimer’s disease (AD) patients, respectively. Elderly and young cognitively healthy (CN) individuals reached aforementioned equilibria earlier. Target and ND regions assumed in curves were composite value from regions with highest ligand uptake (precuneus, posterior cingulate, inferior parietal, and lateral temporal cortices) and cerebellum gray matter, respectively

The stabilization of specific binding (the difference between target and reference region) and total/ND binding (the ratio between target and reference region) was observed at 60 and 90 min for symptomatic individuals, respectively. In young and elderly CN individuals, specific binding and total/ND estimates were low, and the stabilization was reached earlier (Fig. 4).

**Fig. 5:**
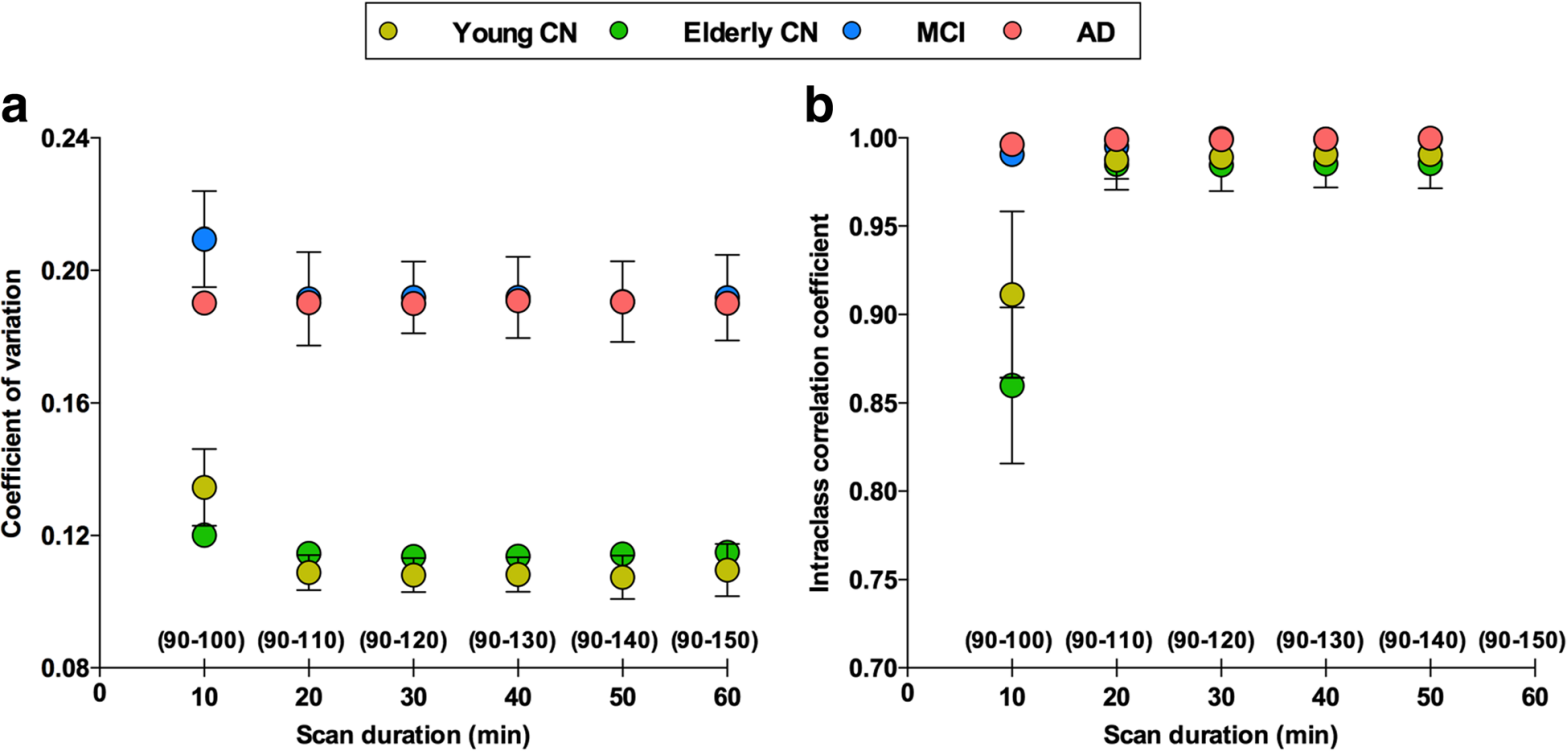
SUVRs measured from 90 to 110 min provide reliable [^18^F]MK-6240 estimates. **a** Dots and bars represent coefficient of variation (CV) and 95% confidence interval (CI), respectively, assessed for each individual’s regions of interest (ROIs) and averaged within groups using standardized uptake value ratios (SUVRs) measured with different durations after tracer reached equilibrium (90 min, see Fig. 4). **b** Dots and bars represent intraclass correlation coefficient (ICC) and 95% CI, respectively, performed between SUVRs calculated using progressively longer frames. 95% CI analyses suggested no differences in SUVR estimates measured using acquisitions equal to or longer than 20 min for [^18^F]MK-6240 scans starting 90 min post injection. CN cognitively healthy, MCI mild cognitive impairment, AD Alzheimer’s disease

The CV and ICC analyses performed after the total/ND binding reached equilibrium indicated that scan acquisitions from 90 to 110 min offer reliable measurements for the [^18^F]MK-6240 SUVR calculation (Fig. 5). Additionally, ICCs between SUVRs measured from 90 to 110 min (SUVR_90–110_) and 2T-CM4k DVRs and BPs (k_3_ / k_4_) were 0.993 (95% CI 0.99–0.995) and 0.791 (95% CI 0.7–0.857), respectively.

**Fig. 6:**
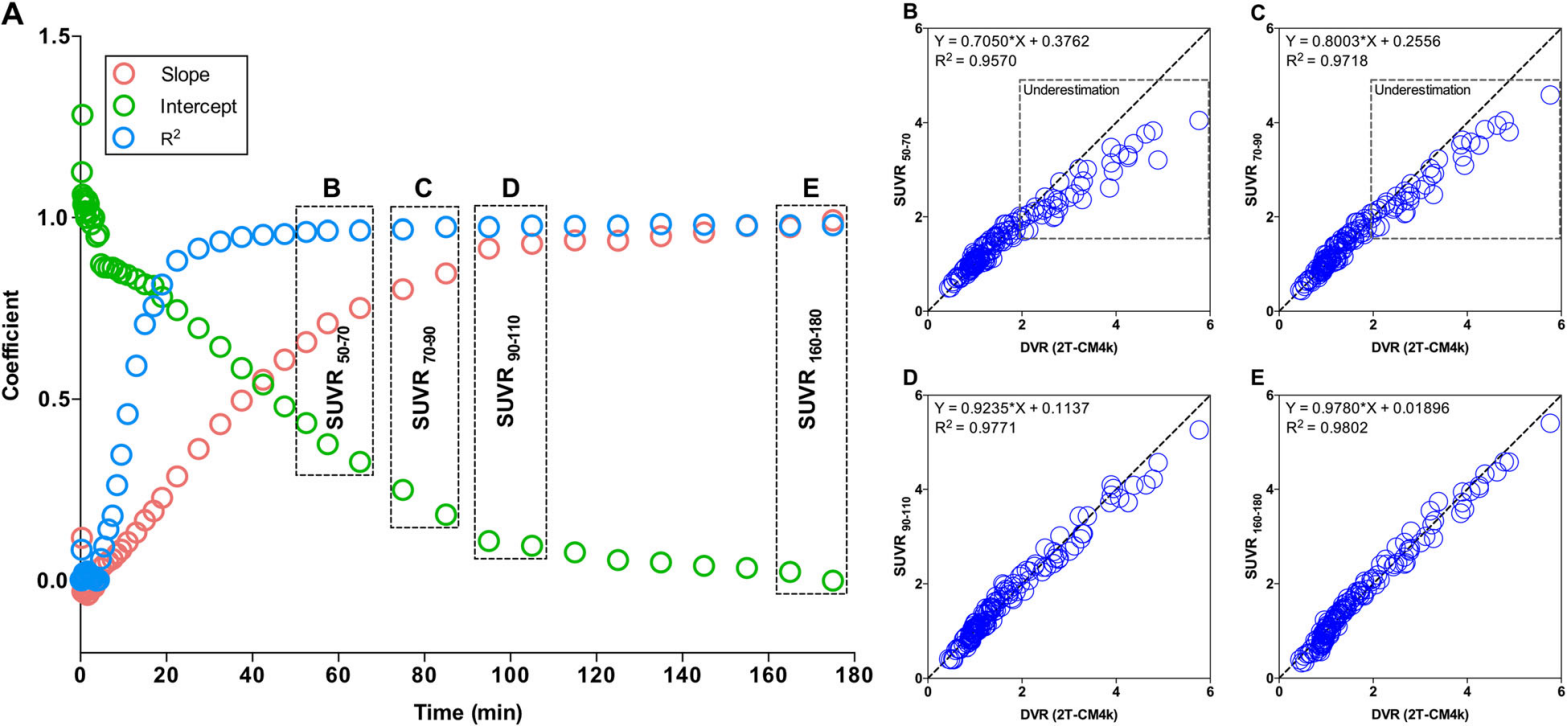
SUVRs measured in later time frames had progressively more similar estimates than compartmental analysis. **a** Dots represent results of regressions between standardized uptake value ratios (SUVRs) obtained from different scan acquisition times and distribution volume ratio (DVR) obtained with reversible two-tissue comportment model (2T-CM4k) across subjects and brain regions. Association between SUVR and 2T-CM4k showed progressively better goodness of fit (*R*^2^) and these quantification methods showed progressively more similar estimates (slope closer to 1 and intercept closer to 0) when using progressively later time frames for SUVR calculation. Although strength of the relationship showed constant increase until end of experiment, it approached the asymptote of the curve at 90 min post injection. Scatter plots show association between 2T-CM4k DVRs and SUVRs calculated from (**b**) 50 to 70 min, (**c**) 70 to 90 min, (**d**) 90 to 110 min, and (**e**) 160 to 180 min. SUVRs calculated before 90 min post injection underestimated 2T-CM4k in regions with moderate and high binding, but not in low binding regions

Using the cerebellar gray matter as the reference, the association between the SUVR and the 2T-CM4k showed progressively better goodness of fit and these quantification methods showed progressively more similar estimates using progressively later time frames for the SUVR calculation, essentially approaching a plateau around 90 min (Fig. 6).

**Fig. 7:**
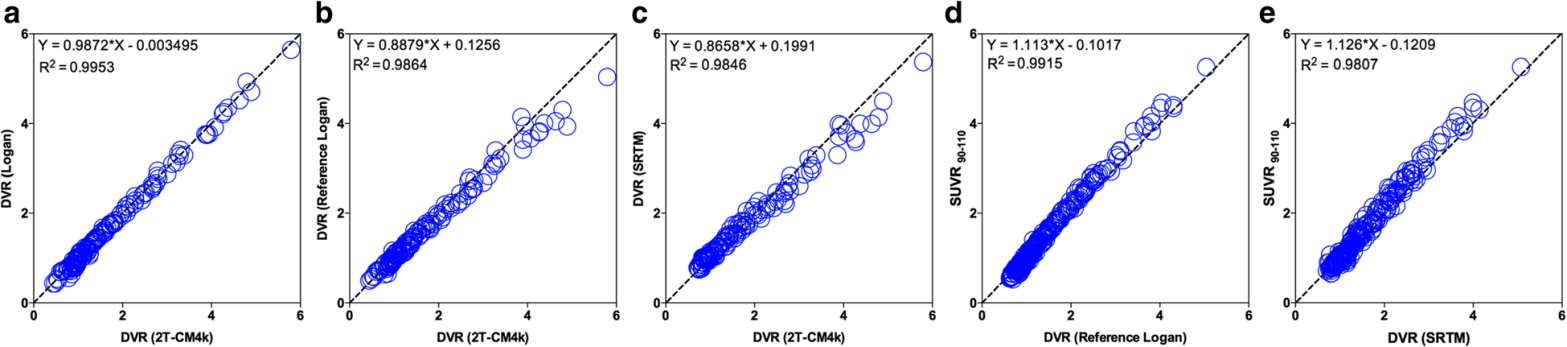
Comparisons between different quantification methods for [^18^F]MK-6240. Scatter plots show regressions performed across individuals and brain regions between (**a**) Logan model vs reversible two-tissue comportment model with four parameters (2T-CM4k), (**b**) reference Logan model vs 2T-CM4k, (**c**) simplified reference tissue model (SRTM) vs 2T-CM4k, (**d**) SUVR_90–110_ vs reference Logan model, and (**e**) SUVR_90–110_ vs SRTM. (a–c) Individuals who underwent arterial blood sampling. (d, e) All participants. DVR distribution volume ratio

In addition, the 2T-CM4k DVRs were highly associated with the DVRs from the Logan method (slope = 0.9872, *R*^2^ = 0.9953; Fig. 7a), the reference Logan method (slope = 0.8879, *R*^2^ = 0.9864; Fig. 7b), and the SRTM (slope = 0.8658, *R*^2^ = 0.9846; Fig. 7c). Moreover, DVRs using the reference Logan method (slope = 1.113, *R*^2^ = 0.9915) and the SRTM (slope = 1.126, *R*^2^ = 0.9807) were highly correlated with SUVR_90–110_ (Fig. 7d, e).

**Fig. 8:**
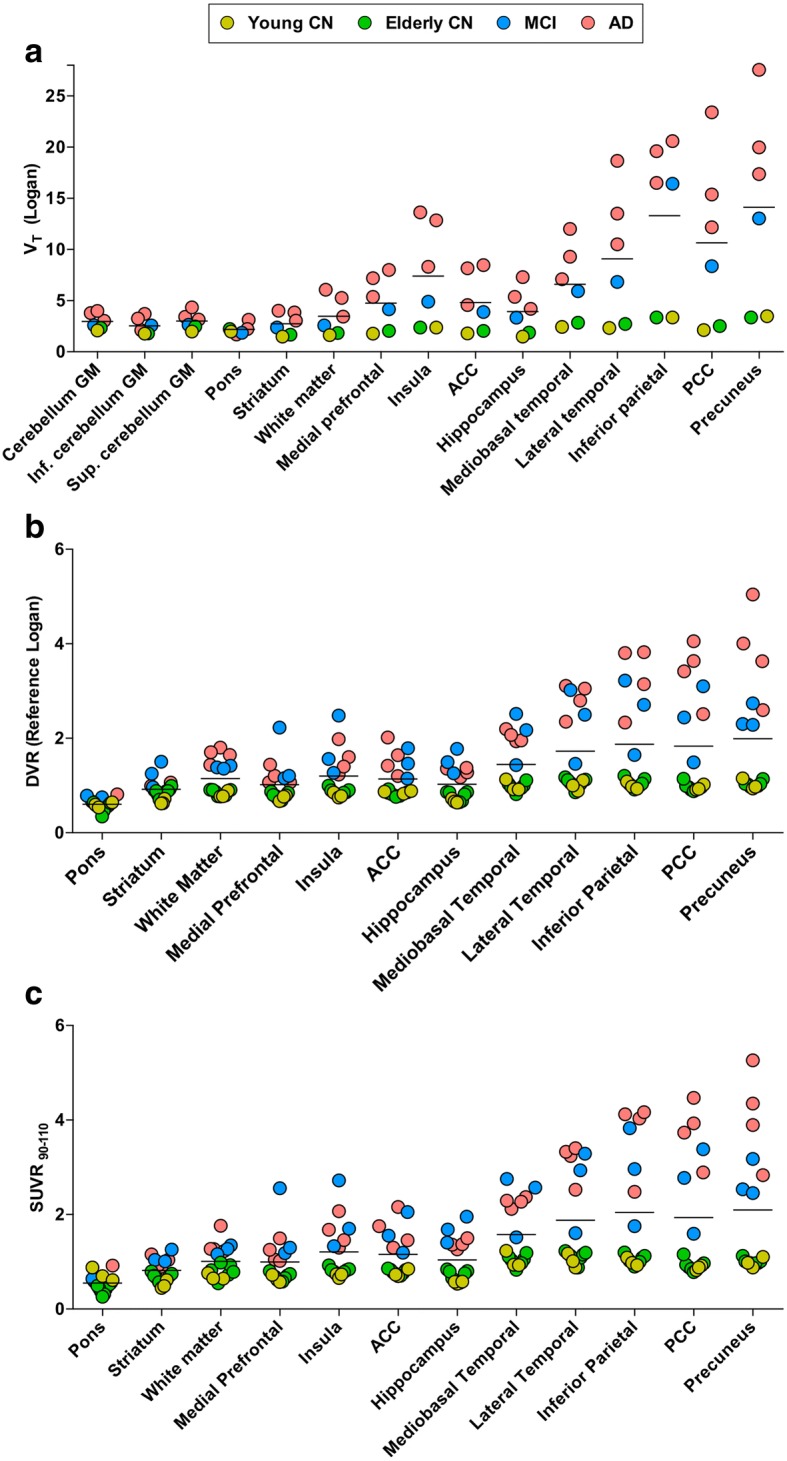
Quantification estimates across clinical diagnosis and brain regions. Horizontal bar represents mean. **a** Total volume of distribution (V_T_; ml/cm^3^) values obtained with Logan graphical method using plasma input function in individuals who underwent arterial blood sampling. **b** Distribution volume ratio (DVR) values obtained with reference Logan method and (**c**) standardized uptake value ratio values measured between 90 and 110 min (SUVR_90–110_) in all individuals, both using cerebellar gray matter (GM) as reference region. CN cognitively healthy, MCI mild cognitive impairment, AD Alzheimer’s disease, Inf. inferior, Sup. superior, ACC anterior cingulate cortex, PCC posterior cingulate cortex

**Fig. 9:**
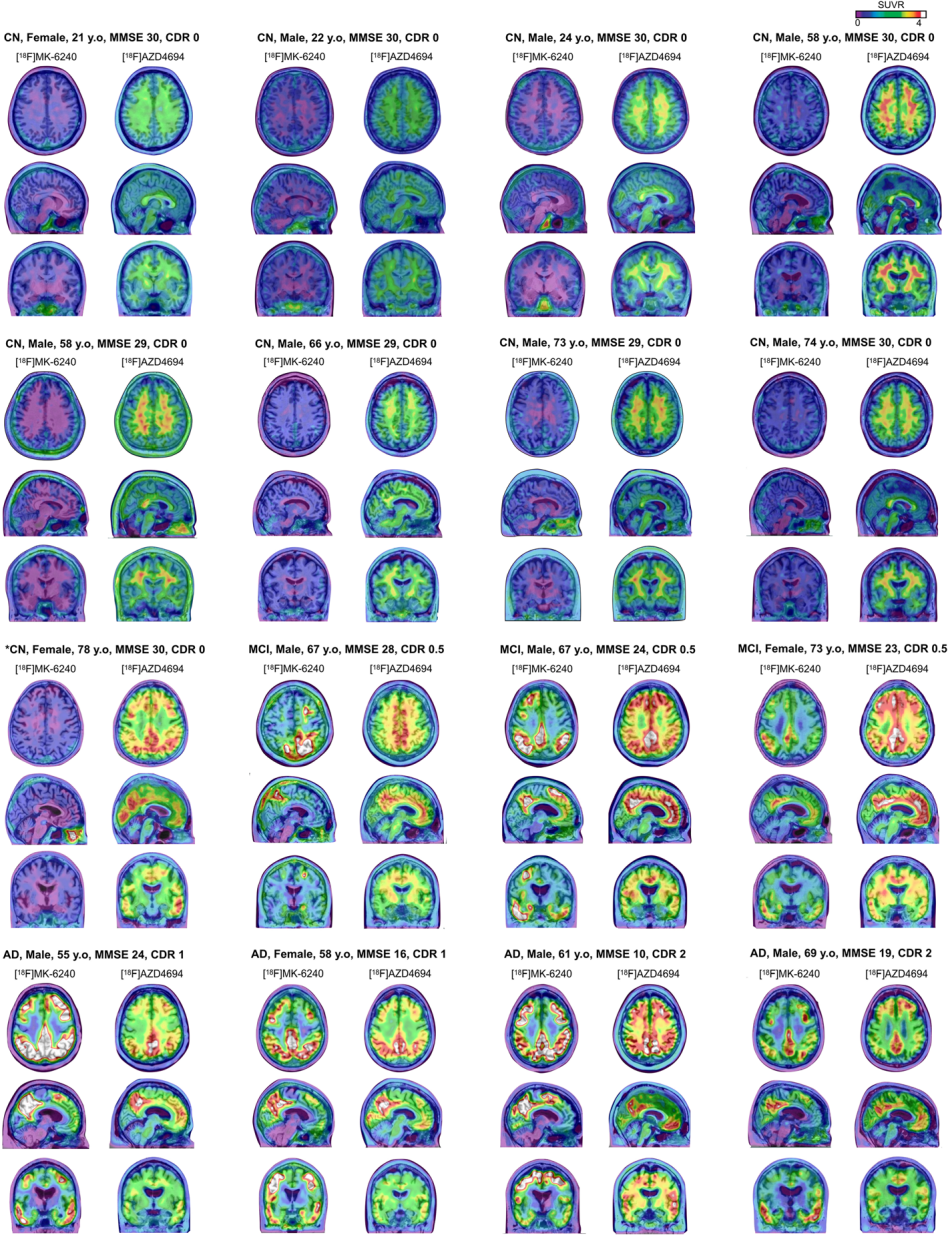
[^18^F]MK-6240 SUVR parametric images of all participants. [^18^F]MK-6240 standardized uptake value ratio (SUVR) averaged between 90 and 110 min and [^18^F]AZD4694 SUVR maps**,** overlaid on the individuals’ structural MRI, of all individuals of the population. [^18^F]MK-6240 images show clear visual differentiation between symptomatic (mild cognitive impairment (MCI) and Alzheimer’s disease (AD)) and asymptomatic (cognitively healthy (CN) control) participants. All AD and MCI patients as well as one CN individual (*) were amyloid-β positive. CDR Clinical Dementia Rating, MMSE Mini-Mental State Examination, y.o years old

2T-CM4k and Logan V_T_ estimates in the cerebellum gray matter and pons were the lowest and had the lowest variance between individuals and across diagnostic groups (Fig. 8a). DVR and SUVR_90–110_ estimates showed a clear differentiation between MCI and AD from CN individuals (Fig. 8b, c). AD and MCI patients showed the highest binding in the PCC and the precuneus, where the binding in MCI and AD participants were on average around four and three times higher than in elderly CN individuals, respectively. [^18^F]MK-6240 and [^18^F]AZD4694 SUVR parametric images from all individuals of the population are presented in Fig. 9.

## Discussion

We have described here the first human evaluation of the novel PET ligand for neurofibrillary tangles [^18^F]MK-6240 using full kinetic modeling and long scan acquisition duration. In this early observation, [^18^F]MK-6240 was able to differentiate CN individuals from patients with MCI or AD, and valid binding estimates were obtained with simplified methods using a reference region such as the SUVR.

[^18^F]MK-6240 showed fast brain penetration and kinetics after the injection, and one metabolite more polar than the parent compound was found. There were no clinically detectable side effects attributed to the [^18^F]MK-6240 injection in our population. The fact that V_T_ values stabilized during the scan acquisition in humans, together with a previous preclinical observation, indirectly supports the notion that there is no radioactive metabolite gradually entering the brain at significant levels [7].

The time–activity curves were well described with the 2T-CM4k, and the 2T-CM4k and the Logan graphical method using the plasma input function had similar estimates to the reference Logan method, SRTM, and SUVR. Moreover, reference region methods were highly correlated with each other. Together, these results further suggest that simplified reference methods offer valid estimates for the quantification of neurofibrillary tangles in the human brain using [^18^F]MK-6240. Importantly, in our analysis, the estimates from reference methods underestimated the 2T-CM4k. Underestimation of the 2T-CM by reference methods has been observed for several other PET ligands such as [^11^C]PIB, [^18^F]AZD4694, and D2 receptor ligands, and the reasons suggested as underpinnings of this underestimation vary across ligands [13, 30–33].

V_T_ values were lowest in the cerebellar gray matter, pons, striatum, and white matter. The lowest variance between cognitively healthy and demented individuals was found in the cerebellar gray matter and pons, suggesting that these two regions have the highest potential to be used as a reference. Importantly, these regions are reported to be relatively unaffected by neurofibrillary tangles in histopathological AD studies [9]. Since the time–activity curves in the cerebellar gray matter were more stable across subjects, which is expected since it is a larger region, this region was chosen as the reference region. In our analysis, the cerebellar gray matter fitted the 2T-CM4k. Similarly, the fact that more than one compartment is needed to describe a reference region has already been observed with other imaging agents for protein aggregates [13, 34]. In the case of [^18^F]MK-6240, it is unclear whether the uptake in the cerebellar meninges may contribute to this finding. Although the BP (*k*_3_ / *k*_4_) values of the cerebellar gray matter overlapped across the diagnostic groups for some of the individuals, symptomatic individuals had a slightly higher V_T_ than CN individuals, suggesting that future studies should assess the [^18^F]MK-6240 binding in the reference regions.

The moment at which the specific binding peaks and the moment at which the target-to-reference region ratio approaches the plateau have been designated as the peak equilibrium [13] and the transient [33] or secular [27] equilibrium, respectively. Both of these parameters of equilibrium have been used for previous PET studies [13, 35, 36]. In the present study, averaged [^18^F]MK-6240 cortical-to-cerebellar gray matter ratio curves showed a constant relative increase, reaching an asymptote after 90 min. Therefore, this parameter suggested 90 min as the earliest time point for the SUVR calculation, while the SUVR stability analysis suggested that there is no benefit in acquisitions longer than 20 min for scans starting 90 min post injection. Taking these observations together, we determined scans performed at 90–110 min post injection to have an optimal tradeoff between duration and reliability.

As a subnanomolar affinity tracer, [^18^F]MK-6240 reached equilibrium earlier (60 min) in low as compared with medium and high binding regions, where the ratio between the total/ND binding stabilized after 90 min. Therefore, SUVRs calculated with frames obtained earlier than 90 min lead to a progressively higher underestimation of tau pathology among low, medium, and high load regions. As a consequence, it is expected that SUVRs estimated earlier than 90 min will underestimate the rate of tau accumulation over time. In the context of anti-tau clinical trials, SUVRs obtained before 90 min will reduce the drug effect size since pretreatment estimates will present a higher underestimation than posttreatment estimates. Studies in other subnanomolar affinity tracers, such as the D2 receptor ligands, have already shown the pitfalls of using early time windows for binding estimation in tracers with distinct times to reach the equilibrium in regions with low and high-density receptors [35].

MCI and AD patients showed [^18^F]MK-6240 uptake across the whole brain cortex, with the highest binding in the PCC, precuneus, inferior parietal, and lateral temporal cortices. Regional [^18^F]MK-6240 bindings measured with arterial input function and simplified reference methods were able to distinguish MCI and AD patients from CN individuals. In vitro autoradiography further supported greater contrast of [^18^F]MK-6240 uptake in AD patients than in age-matched CN individuals across the aforementioned regions. This regional uptake is consistent with the pattern of neurofibrillary tangle deposition that was previously described in postmortem studies of AD and supports [^18^F]MK-6240 as a promising ligand for measuring neurofibrillary tangles in the human brain [9].

Apparent differences in binding sites between [^18^F]MK-6240 and the other available tau tracers were observed in this evaluation. For example, in contrast to the other ligands for neurofibrillary tangles, [^18^F]MK-6240 had minimum uptake in the striatum, which despite being known as a region with low tangles concentration is one of the major binding sites of the other available tau tracers [37]. Moreover, the time–activity curves and quantification methods suggested similarities in [^18^F]MK-6240 retention between young and elderly CN individuals within the associative neocortical brain regions that are expected to contain low tangle density in these populations. Together, these initial results support [^18^F]MK-6240 as a ligand with high brain selectivity to neurofibrillary tangles. It is important to mention that possible off-target binding of [^18^F]MK-6240 was observed in regions including the retina, ethmoid sinus, substantia nigra, and dura mater. However, since some of these findings were not consistent among participants, these observations require further validation.

This study has methodological limitations. The absence of confirmation of the presence of neurofibrillary tangles with specific antibodies is a limitation of our in vitro study. The small sample size is a limitation of our in vivo study. However, the subjects studied here had a wide range of dynamic [^18^F]MK-6240 uptake, which allowed the evaluation of the tracer kinetics in individuals with low and high binding. Future studies should address the test–retest reliability of [^18^F]MK-6240 and definitively confirm whether the [^18^F]MK-6240 radioactive metabolite does not appear in the brain in a significant amount. Since the HPLC has limited sensitivity in detecting low radioactivity counts [34], a further study using a more sensitive method of measuring the parent fraction is underway to completely define the limitations of quantifying [^18^F]MK-6240 using simplified methods. [^18^F]MK-6240 had the highest uptake in the precuneus and PCC. Previous postmortem observations did not relate these brain regions with the neurofibrillary tangles patterns typically found in AD [9]. In contrast, a recent PET study has shown that neurofibrillary tangle accumulation in the PCC is highly associated with AD pathophysiology [38]. This study highlights that the PCC may have been overlooked by postmortem observations that do not traditionally assess neurofibrillary tangles in this region [38]. Although we have focused on the PCC for some of our kinetic analysis, it is important to emphasize the importance of other brain regions such as the medial temporal cortex to the AD pathophysiological process. In addition, although our results suggest [^18^F]MK-6240 binding as an indicator of AD-related tangles, it is important to mention that the association of this binding with the clinical staging and natural history of AD can be determined only by larger longitudinal studies. Since our population was limited to controls and individuals across the AD spectrum, more studies are needed to determine [^18^F]MK-6240 binding properties associated with non-AD tauopathies.

## Conclusions

[^18^F]MK-6240 displayed favorable pharmacokinetics with rapid brain penetration and washout. In this early observation, [^18^F]MK-6240 discriminated MCI and AD from controls using methods with plasma input function or simplified SUVR estimates. [^18^F]MK-6240 is a promising new-generation tau radiotracer with the potential to be employed in the evaluation of disease-modifying therapies and future diagnostic use.

## Abbreviations

2T-CM: Two-tissue compartment model
ACC: Anterior cingulate cortex
AD: Alzheimer’s disease
AIC: Akaike information criterion
CDR: Clinical Dementia Rating
CERAD: Consortium to Establish a Registry for Alzheimer Disease
CI: Confidence interval
CN: Cognitively healthy
DVR: Distribution volume ratio
HPLC: High-pressure liquid chromatography
HRRT: High-resolution research tomograph
MCI: Mild cognitive impairment
MMSE: Mini-Mental State Examination
MNI: Montreal Neurological Institute
MRI: Magnetic resonance imaging
OSEM: Ordered-subsets expectation maximization
PCC: Posterior cingulate cortex
PET: Positron emission tomography
ROI: Region of interest
SD: Standard deviation
SRTM: Simplified reference tissue model
SUVR: Standardized uptake value ratio
V_T_: Volume of distribution
